# Gene organization, evolution and expression of the microtubule-associated protein ASAP (MAP9)

**DOI:** 10.1186/1471-2164-9-406

**Published:** 2008-09-09

**Authors:** Magali Venoux, Karine Delmouly, Ollivier Milhavet, Sophie Vidal-Eychenié, Dominique Giorgi, Sylvie Rouquier

**Affiliations:** 1 Groupe Microtubules et Cycle Cellulaire, Institut de Génétique Humaine, CNRS UPR 1142, rue de la cardonille, 34396 Montpellier cédex 5, France; 2 Groupe Pathologies neurologiques et cellules souches, Institut de Génétique Humaine, CNRS UPR 1142, rue de la cardonille, 34396 Montpellier cédex 5, France

## Abstract

**Background:**

ASAP is a newly characterized microtubule-associated protein (MAP) essential for proper cell-cycling. We have previously shown that expression deregulation of human ASAP results in profound defects in mitotic spindle formation and mitotic progression leading to aneuploidy, cytokinesis defects and/or cell death. In the present work we analyze the structure and evolution of the ASAP gene, as well as the domain composition of the encoded protein. Mouse and *Xenopus *cDNAs were cloned, the tissue expression characterized and the overexpression profile analyzed.

**Results:**

*Bona fide *ASAP orthologs are found in vertebrates with more distantly related potential orthologs in invertebrates. This single-copy gene is conserved in mammals where it maps to syntenic chromosomal regions, but is also clearly identified in bird, fish and frog. The human gene is strongly expressed in brain and testis as a 2.6 Kb transcript encoding a ~110 KDa protein. The protein contains MAP, MIT-like and THY domains in the C-terminal part indicative of microtubule interaction, while the N-terminal part is more divergent. ASAP is composed of ~42% alpha helical structures, and two main coiled-coil regions have been identified. Different sequence features may suggest a role in DNA damage response. As with human ASAP, the mouse and *Xenopus *proteins localize to the microtubule network in interphase and to the mitotic spindle during mitosis. Overexpression of the mouse protein induces mitotic defects similar to those observed in human. *In situ *hybridization in testis localized ASAP to the germ cells, whereas in culture neurons ASAP localized to the cell body and growing neurites.

**Conclusion:**

The conservation of ASAP indicated in our results reflects an essential function in vertebrates. We have cloned the ASAP orthologs in mouse and *Xenopus*, two valuable models to study the function of ASAP. Tissue expression of ASAP revealed a high expression in brain and testis, two tissues rich in microtubules. ASAP associates to the mitotic spindle and cytoplasmic microtubules, and represents a key factor of mitosis with possible involvement in other cell cycle processes. It may have a role in spermatogenesis and also represents a potential new target for antitumoral drugs. Possible involvement in neuron dynamics also highlights ASAP as a candidate target in neurodegenerative diseases.

## Background

Microtubules (MTs) are linear polymers which self-assemble from α-β tubulin heterodimers in the presence of GTP [1-3]. They participate in a number of functions within cells including chromosome movements in mitosis, vesicle and organelle motility, cell polarity, and flagellar-based motility [4-6]. During interphase, MTs are organized in astral arrays that radiate from the centrosome, and function as a scaffold to direct organelle and vesicle trafficking. During mitosis, the centrosome mediates the assembly and organization of the mitotic spindle that is required for correct chromosome segregation.

This variety of roles is made possible by the dynamic nature of the MT cytoskeleton, modulated by multiple accessory proteins [7,8]. A delicate balance between MT stabilizing and destabilizing proteins is believed to generate the MT dynamics observed in cells [9-14]. Molecules regulating MT dynamics have been identified, and their effects on the assembly of purified tubulin into MT determined. Several spindle proteins, including XKCM1 and XMAP215 family members have been identified that either stabilize or destabilize MTs by mediating the rapid changes between polymerization and depolymerization [8,15]. These proteins play an important role in spindle formation. Another important group of spindle proteins comprise motors of the kinesin and dynein families essential for mitotic progression [8,15-18], and the small GTPase Ran [19-21].

MTs are especially abundant in testis and neurons. Cell-type specific MTs, such as the Sertoli cell MTs and the manchette and flagellum MTs of the spermatids, play essential roles in spermatogenesis. Others are important for flagellar-based motility. In neurons, their integrity is essential to maintaining normal neuron morphology and in the transport of materials between cell body and synaptic terminals. The MT network undergoes drastic changes in neural cells at different developmental stages. For instance, during neural proliferation, MTs assemble into the highly organized mitotic spindle on entering mitosis. In order to transmit signals, neurons stop dividing early in development and direct their efforts towards the elaboration of elongated cellular processes. Neurons are thus terminally post-mitotic cells that no longer form mitotic spindles. After the post-mitotic neurons are generated, they extend a directional process and migrate towards their destination, during which another MT network takes place. MAPs have been shown to directly regulates MT dynamics during many of these developmental processes.

We have recently characterized a novel human MAP named ASAP (ASter-Associated Protein) [22] that was renamed MAP9 in the nomenclature. ASAP localizes to MTs in interphase, associates with the mitotic spindle during mitosis, localizes to the central body during cytokinesis and directly binds to purified MTs by its COOH-terminal domain. Overexpression of ASAP induces profound bundling of cytoplasmic MTs in interphase cells and aberrant multipolar or monopolar spindles in mitosis. Depletion of ASAP by RNA interference results in severe mitotic defects, inducing the formation of aberrant mitotic spindle, delays in mitotic progression, defects in chromosome congression and segregation, defective cytokinesis and cell death. We have also shown that ASAP is a substrate of the oncogenic mitotic kinase Aurora-A [23]. The phosphorylated form of ASAP localizes to centrosomes from late G2 to telophase and to the midbody during cytokinesis. Aurora-A depletion induces a proteasome-dependent degradation of ASAP. ASAP depletion provokes spindle-defects that are specifically rescued by expression of a phosphorylation-mimetic ASAP mutant indicating the need for ASAP phosphorylation by Aurora-A for bipolar spindle assembly and mitotic progression. These previous results suggest a crucial role of ASAP in the organization of the bipolar mitotic spindle, mitosis progression and cytokinesis and highlight ASAP as a putative target for cancer treatment.

Mice knock-out studies and *Xenopus *egg extracts have allowed detailed studies providing valuable information about the involvement of genes in regulatory pathways in the first model, and MT, mitotic spindle formation and cell cycle in the latter. In this report, we characterized the structure of the ASAP gene in human and mouse. We identified the ASAP orthologs in different species in order to perform phylogenetic and molecular evolution analyses and cloned and characterized the murine and *Xenopus *orthologs. We also show that ASAP is predominantly expressed in testis and brain, especially in the germ cell line and in the growing neurites, opening up new perspectives on ASAP functions.

All together, these results shed light on new putative functions of ASAP, in its role both in mitosis and post-mitotic neurons, and provide new tools for the scientific community.

## Results

### ASAP orthologs

**Figure 1:**
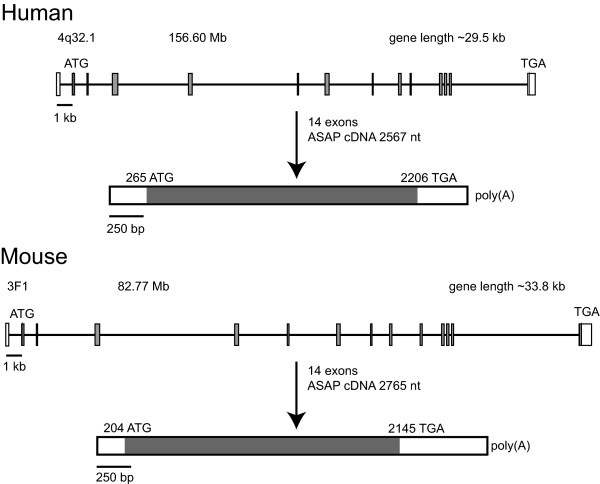
**Comparison of the ASAP gene structure in human and mouse**. The coding regions are represented by horizontal solid lines and the exons by closed boxes. The ATG start codons and the TGA stop codons are indicated, as well as the chromosome locations (cytogenetic bands and nucleotide locations in Mb). The transcribed mRNAs are represented below each gene.

ASAP has been identified as a new MAP. By comparing the ASAP cDNA and its deduced protein sequence with the human genome and the putative transcripts of the Ensembl database, we assigned the ASAP gene to chromosome 4q32.1. The ASAP gene is transcribed on the minus strand, spans ~29.5 kb and contains 14 exons (Fig. 1). BLAST searches identified a conserved potential homolog in mouse (mASAP, 647 amino acids, 71% Protein Sequence Identity or PSI) and partial *Xenopus laevis *(frog) ESTs (Table 1). Comparison of human ASAP with the published genomes of dog (*Canis familiaris*), chicken (*Gallus gallus*), chimpanzee (*Pan troglodytes*) and zebrafish (*Danio rerio*) allowed us to identify ASAP homologs in these species. BLAST searches in translated EST databases using the tblastn option, identified ASAP transcripts in all mammal species including primates, cow, pig, mouse, rat, bat, squirrel, rabbit, cat, dog and elephant, as well as in shrew, opossum, hedgehog, armadillo and platypus. ASAP is also present in birds (chicken), fish (fugu, zebrafish, medaka and stickleback) and frog (*X. laevis *and *X. tropicalis*). We were able to identify the complete protein sequence in mouse, dog (643 amino-acids, >78% PSI), chimpanzee (99% PSI), frog (38.7% PSI) and zebrafish (23.5% PSI), and partial sequences in chicken (>32% PSI). Invertebrate homologs were more difficult to identify. Nevertheless, a distantly related 512-amino acid protein, C34D4.1 (GenBank: NM_068725), that shares ~18.5% amino acid sequence identity and about 35% sequence similarity with ASAP, was identified in the nematode *C. elegans *(Table 1). The highest degree of sequence identity (31%) between ASAP and C34D4.1 is found in the region spanning amino-acids 426–568 of ASAP. Another distant but potential ortholog was found in sea urchin (Table 1), a hypothetical protein sharing 17% PSI and 38% sequence similarity with ASAP (not shown).

**Table 1:** Accession numbers of ASAP orthologs.

**Species**	**GenBank**	**Ensembl**	**Other Databases**
Human	AY_690636, gi :56607126		
	NM_001039580, gi :88759338		
Dog	Predicted XM_850247.1, gi :73978341	Predicted gene ENSCAFG00000008484	
	Prediction XP_855340.1, gi :73978342	Predicted protein ENSCAFP00000012464	
Mouse	NM_001081230, gi :124486996		
	XM_143366, gi :28491857		**XDB**
Frog	EU219608, gi :164633070		AW764964+AW764609
Zebrafish	XM_689238, gi :125803791	Predicted gene ENSDARG00000037276	
		Predicted transcripts ENSDART00000076337 ENSDART00000054225	
		Predicted protein ENSDARP00000054224	
Chicken	Transcript XM_420374.2, gi:118089784	Transcript ENSGALT00000015214	
	Hypothetical protein XP_4203374.2		
		Predicted gene ENSGALG00000020253	
		Predicted transcript ENSGALT00000032288	
		Predicted protein ENSGALP00000031652	
			**Wormbase**
Worm	NM_068725		WP:CE27742
	NP_501126		
Sea urchin	XM_794875.2		

**Figure 2:**
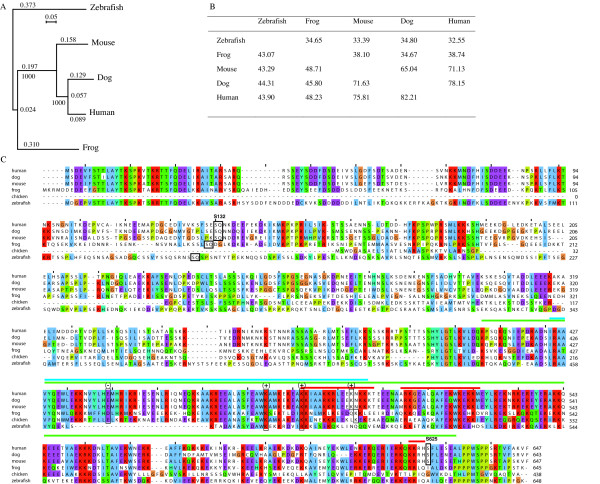
**Phylogenetic analysis and pairwise comparison of ASAP from different species**. **(A) **Phylogenetic tree of the ASAP protein from fish, frog, mouse, dog and human. The predicted protein sequences were compared using the ClustalW software to generate a rooted phylogenetic tree using the bootstrap method. Branch length and bootstrap numbers are indicated. **(B) **Pairwise comparison (%) of ASAP proteins between the species depicted in A. The values above the diagonal show protein sequence identities, those under the diagonal show protein sequence similarities. These values were estimated using the Gap program of the GCG package. **(C) **Clustal alignment of the phylogenetic analysis performed in (A) plus a partial chicken sequence, visualized using Jalview and Clustal X colour codes [63]. On top of the aligment, colour bars indicate conserved domains; blue, MIT-like; green, MAP; red, NLS. Phosphoserines 132 (SQ motif) and 625 are squared. Canonical (+) and (-) residues of the MIT domain are squared.

Accession numbers of ASAP orthologs of selected species used in phylogenetic analyses (Fig. 2). The numbers corresponding to the sequences used in these analyses are underlined. Numerous references correspond to predicted transcripts and proteins. Different databases were searched: GenBank , Ensembl , XDB (*Xenopus *datbases, , Wormbase .

Most vertebrate orthologs are contained in chromosomal regions that are syntenic to human (not shown), i.e., on chromosome 3F1 in mouse (this region is syntenic with human chromosome 4q32.1 as described in , chromosome 3 in chimpanzee (at 174 Mb), chromosome 15 in dog (at 55.9 Mb) and chromosome 4 in chicken (at 21.2 Mb). The *X. laevis *cDNA (645 amino-acids) was assembled using clones AW764964 and AW764609 (Table 1). The zebrafish gene is located on chromosome 1 at 26.02 Mb and has a similar intron-exon structure to that of human (not shown). Using the ASAP protein sequences from selected species we performed a phylogenetic comparison using Clustalw with a bootstrap method. As shown in Fig. 2, the human and dog proteins are very similar (>78% PSI) whereas mouse ASAP is more divergent. Although frog and fish ASAP are more distant than those from the other species, they do not constitute a separate family, since they have a similar topology and share >32–38% PSI and >44–48% sequence similarity with the human protein. Pairwise sequence comparisons confirmed these results (Fig. 2B).

In addition, the multiple alignment used for the phylogenetic analysis revealed two highly conserved regions (about 90% PSI between human, mouse and dog), i.e. the N- and C-terminus ends (amino acids 1–98 and amino acids 420–647, respectively), and a more divergent central region (≤50% PSI). The N-terminus region contains no known motifs or domains, whereas the C-terminus region corresponds to the MAP domain and contains three nuclear localization signal (NLS) motifs (see below) (Fig. 2C).

**Figure 3:**
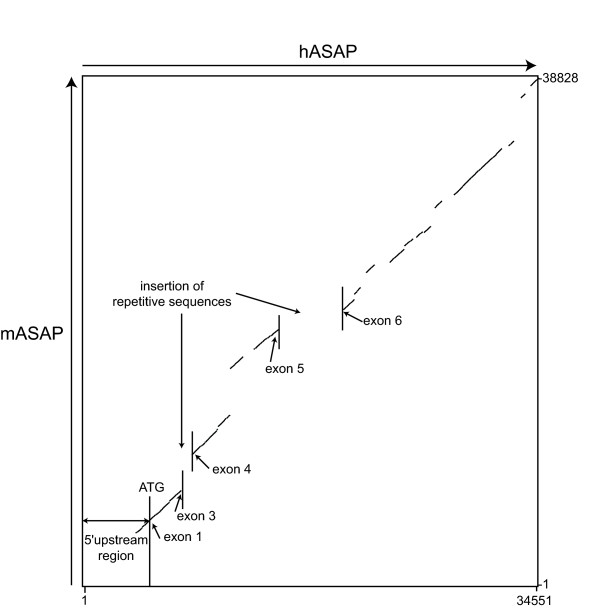
**Dot plot of the percent identity plot (PIP) analysis of human and mouse ASAP gene regions**. The human ASAP sequence (hASAP) is represented on the x-axis, and the mouse sequence (mASAP) on the y-axis. The ATG start codon and the 5' upstream region are indicated, showing that the two genes are conserved for ~1 kb upstream the coding region. Introns differ between the two species by homology losses due to large insertion/deletion of repeats as examplified between exons 3 and 4, and exons 5 and 6.

To follow the evolution of the ASAP gene, we analyzed the mouse gene and compared it with the human ortholog. As shown in Fig. 1, the mouse gene is ~33.8 kb long, localizes on chromosome 3F1 at 88.77 Mb and is also composed of 14 exons encoding a mRNA of ~2.7 kb. The predicted protein is also 647 amino-acids long and shares >71% PSI with human ASAP. The coding exons have the same lengths in both species, except for exons 2 (N-terminus) and 14 (C-terminus) whose non-translated moieties vary (not shown). Exon-intron junctions are identical (same codon and amino acid positions) in both species (not shown). In contrast, the intron length may vary considerably. We next compared human and mouse ASAP using the PipMaker software. The dot plot of the percent identity plot (PIP) indicates the regions of similarity with the mouse sequence and the location of repetitive sequences (Fig. 3). Exons are conserved (≥ 75% on average), whereas introns are more divergent with a nucleotide sequence identity as low as <50% as is the case for introns between exons 3–4 or 5–6, mainly due to insertions or deletions of repetitive sequences in either species. Interestingly, a ~1 kb region upstream of the first codon is conserved in the two species and may therefore contain the promoter region. One weak CpG island (67% GC) was identified in the region 5 kb upstream of the first exon corresponding to nucleotides -1371 to -879 before the ATG start codon (not shown). Numerous transcription factor binding sites including SP1 sites, CAAT, TATA and GC boxes were identified in the region 2 kb upstream of exon 1 using TFSCAN (not shown).

### ASAP domains and motifs

**Figure 4:**
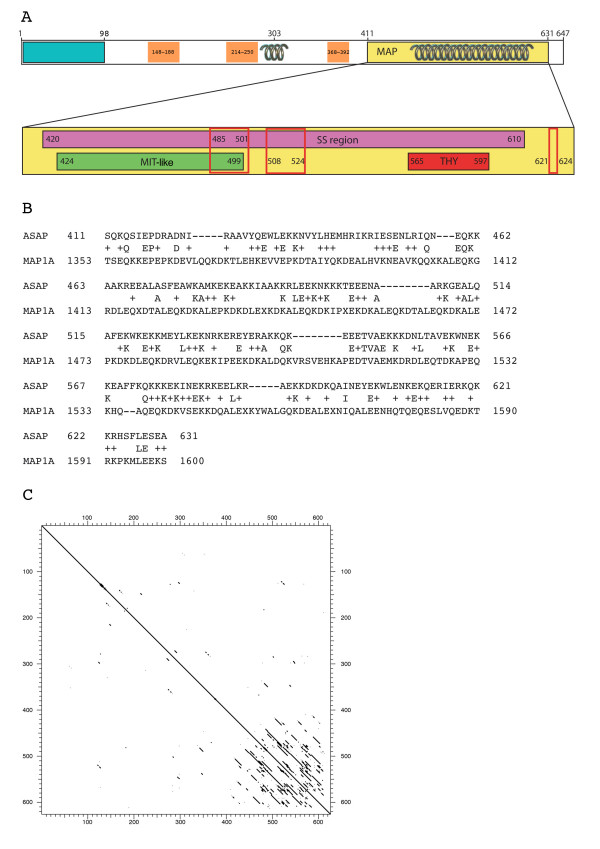
**Domains and motifs of the ASAP protein**. **(A) **Top: General structure of ASAP. The residues are numbered 1 (N-terminal end) to 647 (C-terminal end). The MAP domain is indicated as well as the coiled-coil regions (springs). The blue rectangle indicates the region that is the most conserved during evolution in the N-terminal part (Fig. 2), orange boxes indicate moderatly conserved regions and uncoloured portions represent variable regions. Bottom: Detail of the MAP domain. This domain contains a self similarity (SS) region as MAP1A, an MIT-like and an THY domain. The nuclear localization signals (NLS) are boxed in red. MAP, microtubule-associated protein; MIT, microtubule interacting and trafficking domain; THY, thymosin beta actin-binding motif. **(B) **Comparison of ASAP with MAP1A. Blast comparison of ASAP with protein databases showing a similarity region with human MAP1A containing a high fraction of Glu and Lys (43, 21.8% and 52, 26.4% respectively in ASAP). **(C) **Protein self matrix of human ASAP. The comparison was performed using the Strider software (window 23 amino-acids, stringency 5 amino-acids). One region with self-similarity (SS-ASAP, amino-acids 420–610) corresponding to the region homologous to MAP1A, could be identified.

We next compared the ASAP protein sequence with the databases using different programs. No obvious consensus or known motif was found, except discrete sequence similarities for the C-terminal part (Figs. 2C and 4A). The N-terminal part of the human protein (amino-acids 1–396) contains a conserved region (1–98), moderately conserved regions (148–187, 214–250, 368–392) and variable portions dispersed throughout the 99–396 region. The C-terminal moiety (~410–630) is weakly homologous with various proteins involved in binding microtubules such as dynein (~27% sequence identity, GenBank: AAN35824), kinesin (~26%, GenBank: AAG51044) and MAPs (microtubule associated proteins) (MAP1A ~22%, GenBank: AAB41132) (Fig. 4B). However, we found no obvious consensus sequence reminiscent of particular protein classes in the ASAP protein. The sequence similarity detected with the proteins described above is mainly due to the richness of the ASAP C-terminal region in Lys (K), Glu (E), Arg (R), and Ala (A) amino-acids.

In mammalian MAPs, the tubulin-binding domains are basic regions containing repeats of charged amino acids as is the case for MAP2, MAP4 and tau with a pI of 10 [24-27]. MAP1B binds to microtubules via a basic domain close to its N terminus, containing 19 repeats of the motif KKE [28] and a pI of 9.65. Two regions of self-similarity, referred to as SS1 and SS2, have been described in MAP1A, due to the presence of repeats [29]. Although rich in charged residues, these two regions contain no highly conserved sequence motifs. The SS1 domain (residues 300 to 500), close to the N-terminus, contains 3 KKE motifs with a pI of ~11.00 whereas the SS2 domain (residues 1307–1606) is acidic with a pI of 4.51, an unusual feature for a MT-binding domain. The charged amino-acids are clustered in non-canonical groups of 3–5 residues separated by irregular intervals. We found a weak sequence similarity of the C-terminal end of ASAP (amino acids 411–631), with a region corresponding to the acidic microtubule-binding region SS2 of MAP1A (amino acids 1353–1606), due to the Glu+Lys richness of both regions but without significant conserved motifs (Fig. 4B). Indeed, using a scrambled ASAP sequence generated using RandSeq , we found a significant similarity level in the same region, demonstrating a bias towards the amino acid composition of MAP1A and ASAP in this region rather than conserved motifs. However, given that the microtubule-binding nature of this ASAP region has also been experimentally demonstrated (Saffin *et al*. [22], and below in the "overexpression of ASAP deletion mutants" paragraph), we indicate it as a MAP region in Figs 2 and 4. A dotplot matrix comparison of ASAP with itself identified a region with self-similarity (amino acids 420–610, Fig. 4C), revealed as sharing common features with SS1 with amino acid analysis (Table 2). The two regions are about the same length, have the same charged amino acid composition and are basic (pI ~11), in contrast to SS2 which is acidic with a pI of 4.50. They also share different motifs such as KEE, EKKE and EKKDK(E/D)K.

**Table 2:** Self-similarity regions of ASAP and MAP1A.

	MAP1A		ASAP
	
	SS1	SS2	SS region
Length (aa)	200	300	197
pKi	11.00	4.49	10.72
			
Asp (D)	8 (4%)	33 (11%)	3 (1.5%)
Glu (E)	32 (16%)	52 (17.3%)	43 (21.8%)
Lys (K)	54 (27%)	36 (12%)	52 (26.4%)
Arg (R)	14 (7%)	19 (6.3%)	14 (7.1%)

Comparison of the amino acid composition of MAP1A self-similarity microtubule-binding regions (SS1 and SS2, [29]) with the self-similarity ASAP region (SS).

In addition, PSORTII software identified three NLS in the ASAP C-terminal domain corresponding to amino acids 483–501, 508–524 (bipartite NLS) and 621–624 (monopartite NLS), respectively (Fig. 4A).

**Figure 5:**
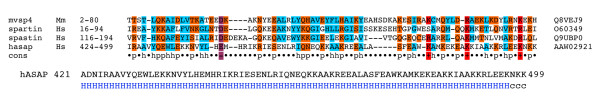
**ASAP possesses an MIT-like domain**. Multiple sequence alignment of selected MIT domain containing proteins [34] with ASAP. The consensus is indicated below the alignment: h, p, + and - indicate hydrophobic, polar, positively and negatively charged residues. Hydrophobic residues are highlighted in blue, polar residues in orange, positive residues in red and negative residues in purple. The secondary structure prediction at the bottom shows that this ASAP region is almost exclusively composed of helix (H). Conserved positions of (+) and (-) amino acids are also indicated in Fig. 2C.

The ASAP secondary structure was predicted using a hierarchical neural network [30,31]. ASAP is mainly composed of alpha helix (~43% in the complete protein, and >72% in the SS region, amino acids 420–610, Fig. 4A). Similar results were obtained using either the mouse or the frog ortholog. The SMART program [32,33] identified two coiled-coil regions spanning amino acids 297–327 and 477–628 (Fig. 4A). Furthermore, a block of ~80 amino acids (420–500) composed exclusively of alpha-helical structures was clearly identifiable from the predicted secondary structure in the SS domain. A search in the PDB database for similar structures, and eye inspection allowed us to identify the MIT domain as possessing similar characteristics [34] (Fig. 5): 3 conserved positively charged residues (+), a unique conserved negatively charged residue and a succession of polar and hydrophobic areas (50% of the positions are conserved, with ~33% of the residues identical or similar to at least one of the aligned MIT sequences). As shown in Fig. 2C, this region is conserved during evolution, and the (+) and (-) amino acid positions are conserved in most orthologs. Furthermore, in the scrambled ASAP sequence, we were unable to identify any MIT-like domain, suggesting that the identification of this domain is not only due to a particular amino acid composition. Therefore, although this region was not identified as an MIT domain using conserved domain search programs, we suggest that this region of ASAP is an MIT-like domain. Although the function of MIT domains remains unclear, one could reasonably assume that they convey the capability to interact with MTs as also described with spastin [35,36]. Another region of the ASAP MAP domain shares sequence similarity with the THY motif (thymosin beta actin-binding motif, Conserved Domain Database: smart00152) with 28% sequence identity (residues 565–597 of ASAP, Fig. 4A).

Finally, as a very rich Ser/Thr protein, ASAP contains several putative phosphorylation sites, among which two are characterized and referenced on : the S625 determined by ourselves and necessary for spindle formation and mitosis completion [23] is conserved only in mammals (Fig. 2C) and the S132 conserved in all vertebrate species as a SQ motif whose activation is typical of the ATM/ATR pathway in response to DNA damage [37]. Other SQ sites are found across the homologs but not significantly conserved.

### Overexpression of Cter and Nter ASAP deletion mutants

**Figure 6:**
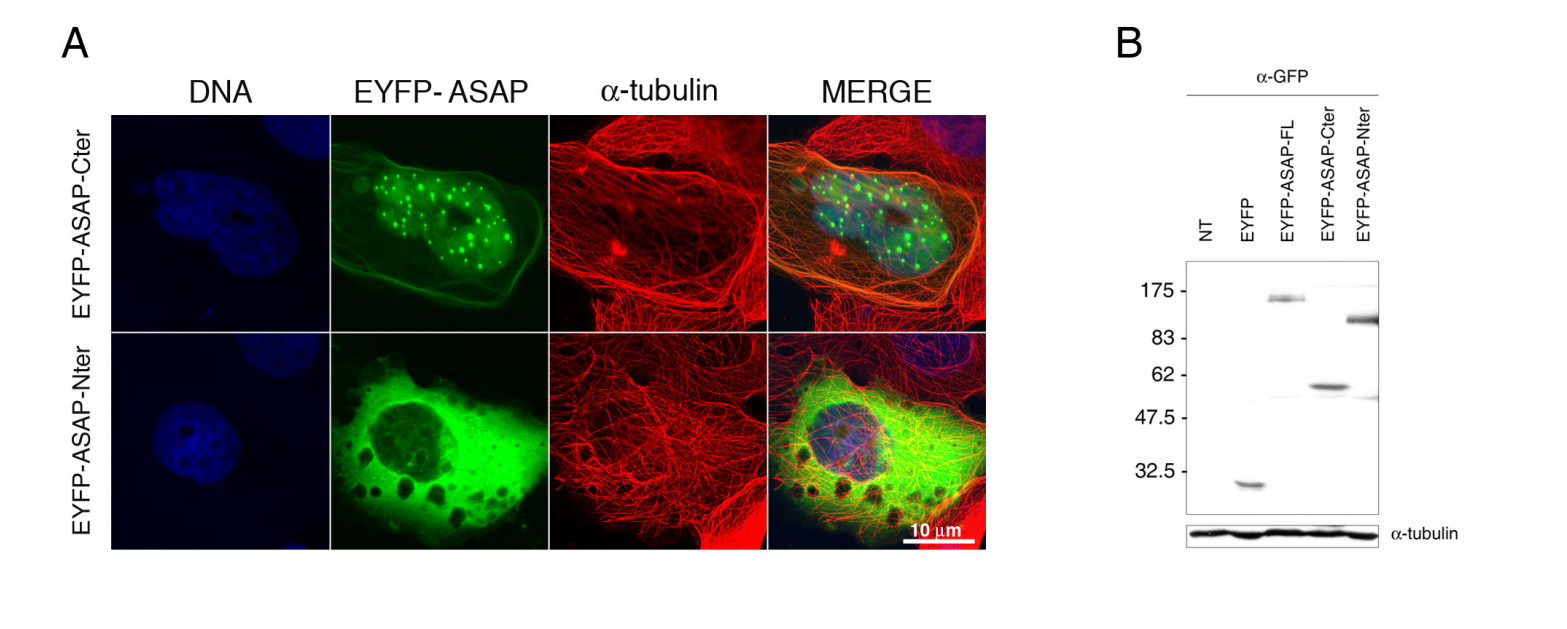
**Overexpression of Cter and Nter ASAP deletion mutants**. Forty-eight hours after transfection with 1 μg of EYFP-ASAP-Cter or EYFP-ASAP-Nter, U2-OS cells were analyzed by immunofluorescence **(A) **and western-blot **(B)**. **(A) **Cells were fixed with PFA and stained with an anti-α-tubulin (red) and Hoechst dye 33258 (DNA). EYFP signals are in green. (Bar, 10 μm). **(B) **Immunoblot of EYFP, EYFP-ASAP-FL (full-length), EYFP-ASAP-Cter or EYFP-ASAP-Nter U2-OS transfected cells using an anti-GFP antibody (top); NT: non-transfected cells. The same blot was probed with an anti-α-tubulin to demonstrate equal loading (bottom).

In order to gain insight into ASAP function and provide support to some sequence analysis data, we overexpressed the Cter and Nter ASAP truncation mutants in U2-OS cell lines (Fig. 6). As already described [22], the EYFP-ASAP-Nter fragment, deleted from its MAP domain led to a loss of the fiber-like distribution in interphase cells, confirming that the MT interaction occurs *via *this CTer domain. However, when this MAP domain was overexpressed (EYFP-ASAP-Cter), the MT-bundling and co-localization could still be observed but the protein appeared also as distinct nuclear foci (Fig. 6A), suggesting that the NLS localized in this domain became accessible. We were unable to determine whether these foci correspond to defined nuclear organelles (not shown). We performed NLS mutations but since they are localized in the MAP domain, the mutants no longer co-localized with the MT network and showed a pattern similar to that observed with Nter domain overexpression (not shown).

### Mouse ASAP characterization

In order to develop the murine model as a tool, we cloned the full-length mouse ASAP (mASAP) cDNA by RT-PCR using total testis RNA and primers derived from the sequence available at the time in the database (XM_143366).

**Figure 7:**
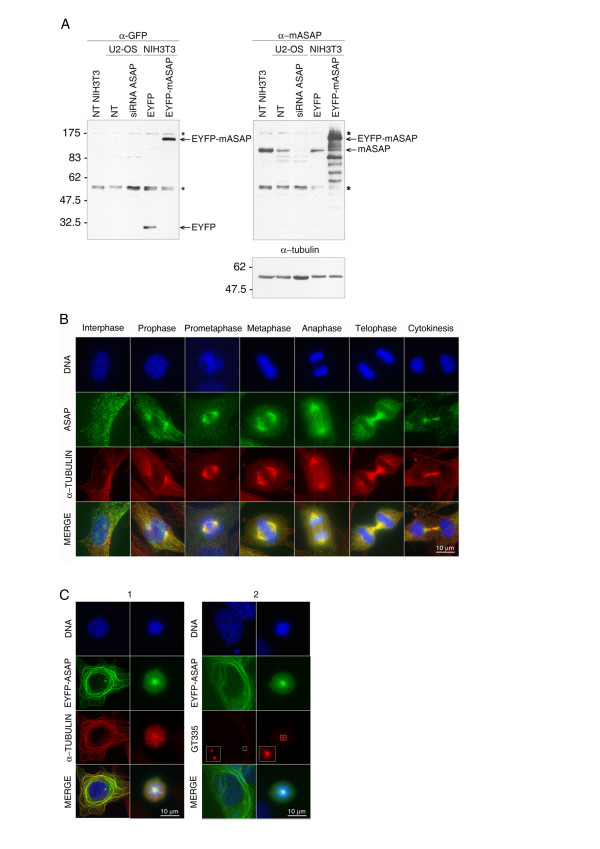
**Characterization of the mouse ASAP**. **(A) **Characterization of anti-mouse ASAP antibodies by immunoblotting. Non-transfected (NT) or transfected U2-OS (with a human ASAP siRNA) or NIH3T3 (with EYFP or EYFP-mASAP) cells were blotted first with an anti-GFP (left), then with the anti-mASAP (right). The specificity of the affinity-purified anti-mASAP antibody was assessed by using cell extracts from NIH3T3 and U2-OS (right), and compared to the profile observed 1) after ASAP-depletion by siRNA in the U2-OS cells (right) and 2) with the anti-GFP antibody against EYFP-mASAP transfected NIH3T3 cells (left). * : non specific bands observed with the α-GFP and that still show up in the α-mASAP blot since the same membrane was used. The same blot was finally probed with an anti-α-tubulin to demonstrate equal loading (bottom). The sizes of molecular weight marker are shown on the left. **(B) **Subcellular localization of endogenous mASAP during the cell cycle. Exponentially growing NIH3T3 cells were fixed with PFA and processed for immunofluorescence with the affinity-purified antibody against mASAP (green), and an antibody against α-tubulin (red). DNA was stained with Hoechst dye 33258 (blue). **(C) **Overexpression of mASAP leads to MTs bundles and monopolar spindle formation in NIH3T3 cells. Forty-eight hours after transfection with 2 μg of EYFP-mASAP, cells were fixed with PFA and stained with an anti-α-tubulin (1) or an anti-GT335 (2) (red) and Hoechst 33258 (DNA). Insets show a higher magnification of GT-335 foci. EYFP signals are in green. (Bars, 10 μm).

We first investigated if mASAP had the same endogenous and overexpression patterns as its human counterpart. We raised polyclonal antibodies against the full-length protein. Affinity-purified antibodies recognized an endogenous single protein band of ~110 kDa in NIH3T3 and U2-OS cells, as observed with human ASAP (Fig. 7A, right). The specificity of the antibodies was assessed by comparing these profiles to that observed with the anti-GFP antibody against EYFP-mASAP transfected cells (Fig. 7A, left), and to ASAP-depleted U2-OS cells by RNA interference (Fig. 7A, right). The endogenous signal disappeared in siRNA-transfected cells and the anti-mASAP recognized the highest molecular form of EYFP-mASAP (~137 KDa, highlighted by the EYFP-antibody) in the corresponding NIH3T3-transfected cells. It is noteworthy that, as suspected by sequence comparison, the mouse antibody is able to recognize the human protein. Unfortunately, as observed for the human antibodies, the two antibodies generated against mASAP gave a significant background in immunofluorescence studies. The same ASAP localization was observed using several fixation methods in U-2 OS (not shown) and NIH3T3 cell lines. We observed an identical subcellular localization between endogenous mASAP and human ASAP. Mouse ASAP co-localizes with the MT network in interphase. During mitosis it first relocates to the poles at the astral MTs and the spindle during prometaphase, metaphase and early anaphase, and then to centrosomal MT asters and the central spindle during anaphase and early telophase. During late cytokinesis mASAP colocalizes with the midzone MT bundles when it is also located on MTs nucleated from centrosomes (Fig. 7B).

We then overexpressed the full-length EYFP-mASAP in U-2 OS (not shown) and NIH3T3 cells. As observed for human ASAP, the overexpressed mASAP induced bundles of stabilized microtubules in interphase cells and the same mitotic defects (i.e. an increased mitotic index with more than 70% of monopolar spindles) [22] even in the fibroblast-derived NIH3T3 cell line (Fig. 7C).

### Tissue expression

**Figure 8:**
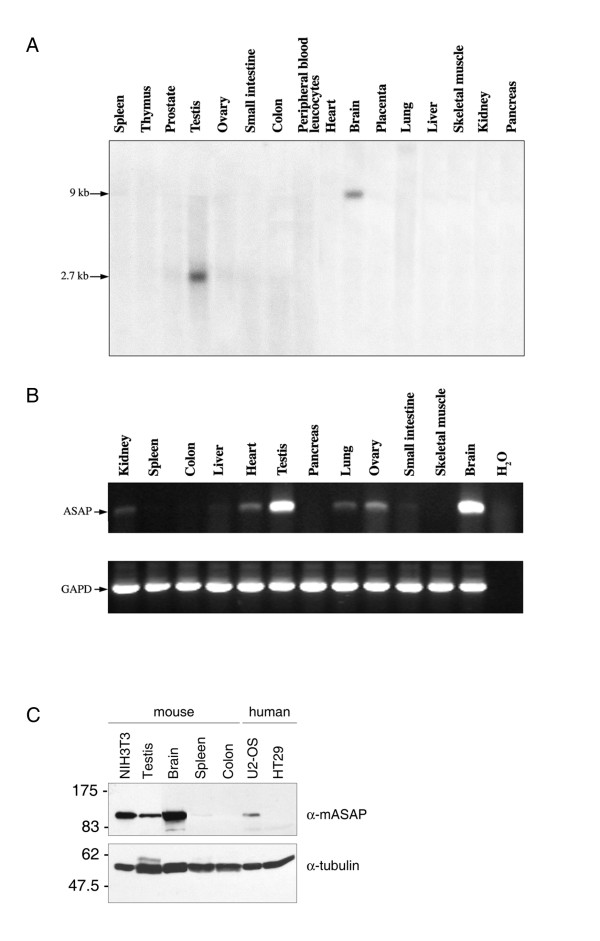
**Expression of ASAP in human and murine tissues**. **(A) **Northern-blot analysis of ASAP transcript in adult human tissues was carried out with pre-made Northern blots purchased from Clontech. The blots were hybridized with a ^32^P-labelled full-length ASAP cDNA probe. The sizes of molecular weight marker are shown on the left. **(B) **RT-PCR analysis of ASAP in murine tissues. Total RNA was prepared from various mouse tissues. A 279-bp ASAP RT-PCR product is visualized. Expression of GAPD was determined as a control of RNA integrity. **(C) **Western-blot analysis of mASAP protein was carried out using the mASAP antibody on testis, brain, spleen and colon mouse tissue extracts and compared to the profile observed on NIH3T3 and U2-OS (positive controls) or HT29 (negative control derived from colon) cell extracts. α-tubulin levels served as loading control.

To investigate ASAP expression, human multiple-tissue Northen blots were probed with an ASAP cDNA probe. A single ~2.6 kb transcript was detected in testis and a ~9 kb one in brain (Fig. 8A). No detectable signals were found in other tissues. To confirm these results, semi-quantitative RT-PCRs were performed on mouse total RNA. ASAP was found to be strongly expressed in testis and brain and to a lesser extent in heart, lung and ovary (Fig. 8B). Supporting these observations, the ASAP transcript is well represented among ESTs derived from different parts of the brain and in testis libraries.

We then investigated if the protein was expressed in brain and testis extracts. As shown in Fig. 8C, ASAP protein is also strongly expressed as a 110 KDa protein in both tissues as observed in NIH3T3 and U2-OS cell line extracts. No expression was found in spleen or colon tissues, or in the colon-derived HT-29 cell line. It is noteworthy that in brain no higher molecular weight protein was detected, suggesting that the 9 Kb mRNA probably corresponded to a partially spliced ASAP mRNA.

**Figure 9:**
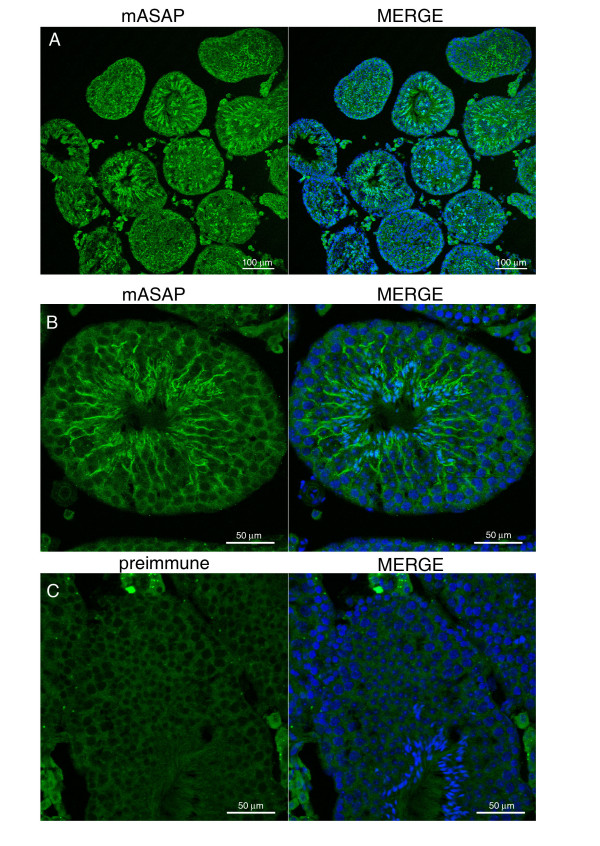
**ASAP expression in testis**. Immunohistochemical staining of testis cross-sections of adult mouse using ASAP-antibody **(A, B) **or preimmune serum **(C)**. Nuclei are stained with Hoechst 33258. Scale bars: 100 μM (A) and 50 μM (B, C).

Since hASAP is involved in mitotic spindle formation, we were not surprised to find the ASAP gene highly expressed in a proliferative tissue such as testis. To determine whether ASAP expression is indeed germ cell line specific, we used *in situ *hybridization. In mouse testis, each seminiferous tubule stage contains a number of spermatogenic cells at different stages of development. Unfortunately, despite different trials of fixation, our antibodies proved not suitable for immunohistochemistry experiments. However, as shown in Fig. 9A–B, we were able to confirm ASAP immunoreactivity observed in seminiferous tubules as a specific cytoplasmic filamentous staining when compared to the preimmune serum (Fig. 9C). We were unable to observe a real co-staining with α-tubulin (not shown). ASAP expression may be stage specific since the hybridization is somehow stronger in some tubules but an adequate antibody would be necessary to confirm this.

**Figure 10:**
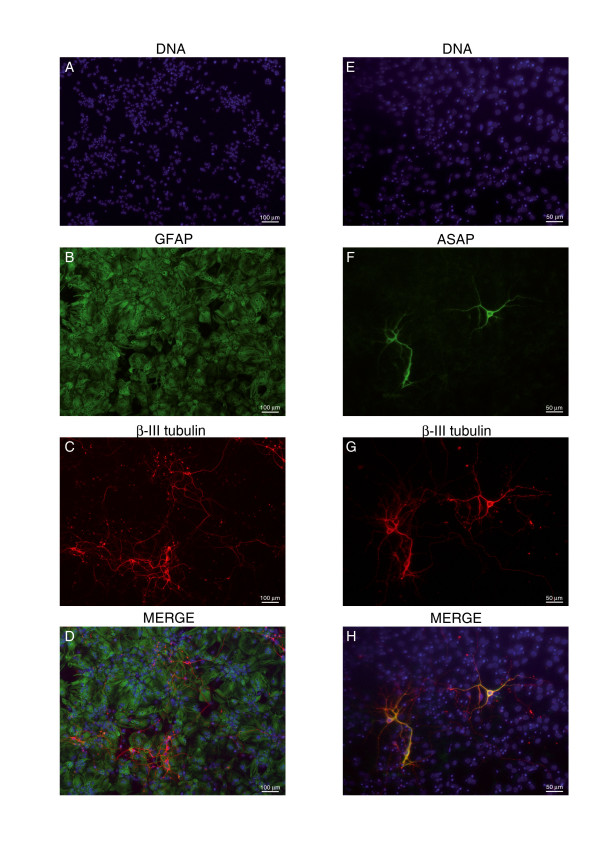
**Expression of ASAP in differenciated neural stem cells**. **(A-D) **After 9 days of differentiation neural stem cells differentiate into neurons and astrocytes. **(A) **Nuclei stained with DAPI (blue), **(B) **astrocytes revealed by GFAP immunostaining (green), **(C) **neuronal cells identified by detection of β-III tubulin (red), **(D) **merged visualization of A-C. **(E-H) **ASAP is expressed in neurons but not in astrocytes after 9 days of differentiation of neural stem cells. **(E) **Nuclei stained with DAPI, **(F) **detection of ASAP, **(G) **β-III tubulin positive neuronal cells, **(H) **merged visualization of E-G. Scale Bar: (A-D), 100 μm; (E-H), 50 μm.

In contrast, normal adult brain is a tissue containing mainly non-proliferating cells. To analyze the expression of ASAP in the brain, we performed immunofluorescence on culture neural stem cells (NSC). In culture, these multipotent cells self-renew, and after mitogen withdrawal, differentiate into neurons, astrocytes and oligodendrocytes in predictable proportions. Here, we addressed the question of whether ASAP is expressed during differentiation of NSC as a first step to studying the possible role of ASAP during brain development. Mouse cortical neural stem cells were isolated from CD1 mouse embryos at 13.5 embryonic days. Nine days after differentiation, cells could be identified by immunocytochemistry as neurons (β-III tubulin positive cells) or astrocytes (GFAP positive cells) (Fig. 10A–D). We also analyzed the pattern of expression of ASAP by immunocytochemistry. ASAP was clearly detected in neuronal cells as demonstrated by co-labeling of cells with β-III tubulin and ASAP antibody (Fig. 10E–H). ASAP was not detected in GFAP positive cells. Therefore in this model, ASAP is specifically expressed in the cell body and growing neurites of post-mitotic neurons. Further experiments are necessary to characterize the function of ASAP in brain.

### *Xenopus *ASAP characterization

**Figure 11:**
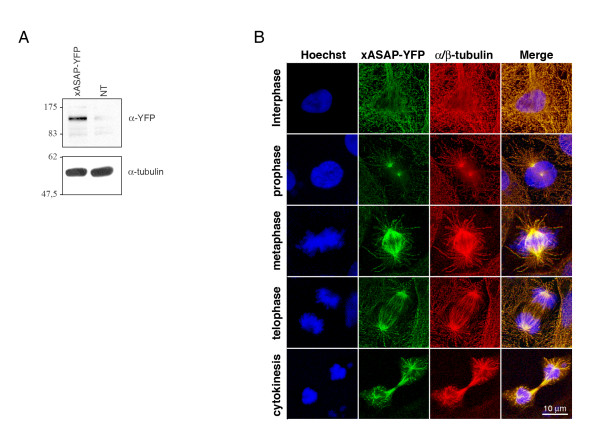
**Characterization of *Xenopus *ASAP**. Asynchronous XL2 cells were transfected with the EYFP-X-ASAP cDNA and analyzed **(A) **by immunoblot using an anti-GFP antibody or **(B) **by immunofluorescence. Cells were fixed in PAF/MTSB and co-stained with the anti α-tubulin (red) and Hoechst 33258 (blue).

In order to develop *Xenopus* as a model system, we PCR-screened a *Xenopus *tadpole stage 24 library using primers derived from EST databases. Only one partial cDNA clone containing the N-terminal part of the sequence was isolated. The full-length clone was PCR-reconstructed using an overlapping cDNA found in the database (see methods). Given the sequence divergence between human ASAP and X-ASAP, we overexpressed the full-length EYFP-X-ASAP in *Xenopus *XL2 cells to observe the subcellular localization. Western blot analysis revealed an expression of EYFP constructs at ~120 kDa, a molecular weight slightly lower than that observed for hASAP or mASAP (Fig. 11A). As observed for mammalian ASAP, overexpressed X-ASAP colocalized with interphasic and mitotic MTS, showing the same localization as both mASAP and hASAP (Fig. 11B). However, we observed neither bundles nor monopolar spindles. Since the transfection level was very low, it was quite difficult to observe transfected mitosis. This may be accounted for by abnormal mitotic cells having undergone cell death.

## Discussion

In a previous report, we demonstrated that ASAP/MAP9 is a novel microtubule-associated protein required for a proper cell cycle progression [22,23]. In this paper, we detected homologs in all vertebrate species investigated and potential orthologs in different invertebrates, thus suggesting a requirement of ASAP in higher eukaryotes. The coding sequence and exon-intron structure have been conserved during vertebrate evolution, suggesting that selective constraints are exerted on this gene to maintain its function. These genes are also invariably located in regions syntenic to the human locus, demonstrating common ancestry. Invertebrate and vertebrate ASAP also likely derive from a common ancestor but which evolved independently after the separation of the two clades. This would account for the low level of homology where events such as insertion/deletion or exon shuffling shaped the ASAP gene differently.

The highly N-terminal conserved region 1–98 (Fig. 2C) corresponds to no known conserved motif and its function remains to be elucidated.

Importantly, the C-terminal MAP region is the most conserved not only within vertebrates, but also between invertebrates and vertebrates, suggesting that the conservation of the MAP function is essential. Indeed, the sequence similarity with C34D4.1 (*C. elegans*) or with the sea urchin protein (Table 1) is higher in the MAP region (amino-acids 428–568 and 340–680, respectively). C34D4.1 could be involved in the regulation of microtubule dynamics since it contains stathmin domains [38] that are known to be involved in the regulation of microtubule skeleton by acting on microtubule dynamics. In the vertebrate MAP region, other motifs such as NLS or an MIT-like domain are also conserved (Fig. 2C).

The sequence similarity of ASAP with different proteins/domains is concentrated in the C-terminal MAP domain and seems connected to microtubule binding/dynamics properties. For example, the character of ASAP as a microtubule binding protein is also found with the structural domains that ASAP shares with MAP1A. In particular there is a striking analogy with the self-similarity region SS1 that is involved in binding microtubules [29]. Besides MAP1A, ASAP is also weakly homologous to dynein and kinesin, two motor proteins that bind to MTs, although no motor activity was found in ASAP (not shown). On the other hand, THY has been shown to be involved in the cytoskeleton organization by binding actin monomers and thus inhibiting actin polymerization. We also identified a potential MIT-like domain in the MAP domain of ASAP. This domain was first described in proteins involved in MT binding and in intracellular transport and named MIT for it being "contained within microtubule-interacting and trafficking molecules". For example the MIT domain has been identified in spastin (responsible for the dominant form of spastic paraplegia) [36], spartin (recessive form of spastic paraplegia) [39], both proteins that interact with microtubules, or VSP4 [40] which is involved in the intracellular protein transport machinery and the regulation of membrane association of several proteins. As with spastin, ASAP overexpression causes perturbations in the MT network. The identification of related MIT domains in ASAP, spastin and spartin may therefore suggest the possible involvement of ASAP dysfunction in pathways leading to pathogenesis.

Many MAPs regulated by Aurora-A and involved in spindle assembly (TPX2, NuMA, RHAMM, TACC3) are nuclear in interphase and recruited after reorganization of the different compartments (nuclear envelope, endogenous membrane structures). They are regulated *via *their NLS by the small GTPase Ran, that releases and activates them from bound importins [19,41]. The presence of these conserved NLS may suggest the regulation of ASAP by this pathway. In our fixation conditions, we observed no ASAP in the nucleus accounted for either by a very weak signal or by the fact that our antibody did not recognize the nuclear epitopes. However, when we overexpressed the Cter domain, we observed a localization of ASAP as nuclear foci, suggesting that the NLS are functional and become accessibles in this mutant. However, these nuclear foci could also suggest that ASAP, under certain conditions that need to be determined, has a nuclear function. Indeed, these foci are reminiscent of those observed after DNA damage. Although a putative BRCT-domain hit (amino acids 66–303) found in the ASAP protein sequence could not be confirmed with confidence, it may nevertheless be indicative of a putative role of ASAP in DNA damage, since BRCT domains are usually found in proteins involved in the checkpoint DNA-damage response. On the other hand, the S132, which is conserved in all vertebrates, corresponds to a SQ motif and has been identified in a large-scale proteomic analysis of proteins phosphorylated in response to DNA damage on consensus sites recognized by ATM and ATR [37]. These data hint towards ASAP playing a role in the DNA damage response.

ASAP is very rich in Ser and Thr residues suggesting multiple putative phosphorylation sites. The S625 phosphorylated by Aurora-A is necessary for spindle assembly and completion of mitosis, but is conserved only in mammals (Fig. 2C) as the emergence of a new feature.

We have cloned the murine ortholog of ASAP and confirm an intracellular pattern similar to its human counterpart. We also showed that deregulation of this protein leads to the same mitotic defects even in the fibroblast derived NIH3T3 cell line, confirming that the phenotypes observed in U2-OS cells were not due to the transformed status of these cells. We have also cloned the *Xenopus *ortholog and demonstrated a similar localization when overexpressed. The identical subcellular ASAP localization within the MT network between these species suggests an evolutionary conservation of MAP function. However, the mitotic defects were not observed in the *Xenopus *cell line because of a lack of transfected mitosis, due either to low transfection efficiency or to the lethal issue of these transfected mitotic cells.

Tissue expression analyses have shown that ASAP is predominantly expressed in testis and brain. Such a distribution pattern has already been described for different MAPs. For example, MAP2 is neural-specific but is also expressed as a lower molecular weight isoform in the testis [42]. The testis is one of the most abundant sources of MT networks. These include mitotic and meiotic spindles, the spermatid manchettes and axonemes, and the Sertoli cell cytoskeleton. Some MAPs, such as E-MAP-115, are required for spermatogenesis. Since ASAP is also expressed in the ovary and is involved in the mitotic spindle formation of cultured cells [22], its strong expression in this proliferative tissue is not surprising and a role in the meiotic spindle could be possible. We have indeed confirmed in a preliminary experiment that ASAP is specifically expressed in the germ cell line during spermatogenesis, and its expression may be stage-specific. One might expect an expression in spermatogonia which undergo rapid successive divisions or in spermatocytes where meiosis takes place. Spermatogenesis is an intricately regulated morphogenetic process during which many structural changes are necessary to produce mature spermatozoa. Differentiation and polarization of the round spermatid are associated with new microtubular configuration, that resembles that of pachytene spermatocytes [43]. On the other hand, ASAP could be present in the perinuclear theca which is a rigid cytoskeleton that covers the entire nucleus of mammalian spermatozoa, and could coat the acrosomal vesicle of round spermatids before attachment to the anterior region of the nucleus. Different MAPs, such as Ndel1 [44], E-MAP-115 [45], MAP4 [46], and tau [47], are present in the spermatid. Together with these proteins, ASAP could be involved in nuclear shaping and the process of spermatid elongation. Deciphering the exact location of ASAP expression will require antibodies adapted for immunohistochemistry experiments.

Microtubules are also essential for a number of cellular processes that include the transport of intracellular cargo or organelles across long distances. They are especially abundant in neurons, where they exhibit an extreme state of stability. They are involved in neuronal migration and positioning during cortical development. After the post-mitotic neurons are generated, they extend a directional process and migrate towards their destination, during which another MT network takes place. MAPs have been shown to be the direct regulators of MT dynamics during many of these developmental processes. In neurons, the major MAPs include tau, MAP1A, MAP1B and MAP2. These MAPs are phosphoproteins and the level of phosphorylation has been shown to regulate their activities to stimulate MT assembly. Tau has been associated with different neurodegenerative diseases such as Alzheimer's disease, Pick's disease and frontotemporal dementia associated with Parkinson's disease [48,49]. However, several other proteins such as Ndel1, the partner of LIS1 involved in lissencephaly [50], ASPM involved in microcephaly [51], or spastin involved in hereditary spastic paraplegia [52], are also neuronal MAPs. The identification of numerous MAPs and the progressive elucidation of the mechanisms of MT assembly and transport are beginning to have a profound impact on the study and treatment of human genetic diseases such as neurodegenerative diseases (Huntington's disease, Alzheimer's disease) (for review see [53]). Here we have demonstrated the specific expression of ASAP in neurons and growing neurites, suggesting an important role in the brain. It is noteworthy that neuronal MAPs described above such as Ndel1, spastin and ASPM are also expressed at the mitotic spindles of cell cultures [36,54,55]. Even though neurons are quite dissimilar from typical interphase cells with regards to MT distribution and organization, several observations suggest that axonal and dendritic arrays may be established by mechanisms very similar to those used for the formation and function of the mitotic spindle [56].

Adult CNS neurons are considered as postmitotic but it appears that these cells must keep their cell cycle in check to avoid any reinitiation leading to an altered state. There is now growing evidence that neurons at risk of neurodegeneration are also at risk of reinitiating a cell cycle process [57], and several neurodegenerative disorders are related to cell cycle failures. In human, cell cycle events (loss of cell cycle control) are associated with several neurodegenerative diseases such as Alzheimer disease and ataxia telangiectasia [58].

## Conclusion

ASAP is a novel MAP whose expression defects provoke aberrant mitoses leading to cell phenotypes reminiscent to those observed in cancers. ASAP is phosphorylated by the oncogenic mitotic kinase Aurora-A that plays a key role in mitotic spindle formation and the cell cycle, highlighting ASAP as a potential new target for anti-tumoral drugs [22,23]. In this work, evolutionary and expression studies have shed light on new putative functions of ASAP both as a germ cell line and neuronal MAP that could be involved in spermatogenesis and neuronal developmental processes. Consequently, deregulation of ASAP expression in such tissues, as observed with other MAPs, may lead to spermatogenesis defects or neurodegenerative disease. Although our analysis shows an evolutionary conservation of MAP function in ASAP, it also suggests also that this protein might be involved in other cell cycle processes such as DNA damage response. Our data also validate mouse and *Xenopus *as models for further ASAP studies using either knock-out or MT *in vitro *experiments.

## Methods

### Sequence analysis

We performed general database searching using the BLAST program of the GCG package (University of Wisconsin) [59]. Potential coding regions and gene structure were identified by comparison of the cDNA sequences with the genome sequences and the predicted transcripts , and examination by eye. We performed sequence comparisons using the Clustalw v. 1.8 software package [60] and PipMaker [61]. Clustalw was used for the phylogenetic analysis and the tree was constructed using the neighbor joining method [62], based on the number of amino acid substitutions. The numbers on internal branches represent the frequency of occurrence among 1000 trees (bootstrap method with the Clustalw package). Clustal alignment was visualized using Jalview and clustal X colour codes were used as defined in the Jalview options [63]. PipMaker was used for determining local alignments of human and mouse genes. Gap-free segments are displayed in a PIP (percent identity plot) and the corresponding dot-plot. We searched CpG islands using the cpgplot software from EMBOSS [64]. Transcription factor binding sites were searched using TFSCAN from EMBOSS  and promoter regions were searched using the neural network promoter prediction software .

Protein domains and motifs were searched using the following programs and databases: phi- and psi-blast programs of the NCBI platform, SMART [32,33], prosite , pfam , MotifScan , PSORTII , all softwares being available in the Expasy package .

Secondary structures was predicted using a hierarchical neural network (HNN [30,31], )

### Cter and Nter ASAP deletion mutants sub- cloning

The truncation mutants EYFP-ASAP-Cter (420–647) and EYFP-ASAP-Nter (1–420, that corresponds to the EYFP-ΔCter described in [22]) were constructed in pEYFP-C1 (Clontech, EYFP in N-ter) and pEAK-EGFP ((EGFP in C-ter). Since the same patterns were obtained in overexpression, only the EYFP constructions are presented in the results section.

### Mouse and *Xenopus *cDNA cloning

BLAST analyses revealed a full-length mouse cDNA clone in the databases. We obtained the mouse ASAP cDNA (mASAP) by RT-PCR using total testis RNA and primers derived from the mouse sequence (mASAP-1F: 5'-ATGTCCGATGAAATCTTCAGCAC-3' and mASAP-1R: 5'-AAATACTTTTGAGGGCGCAGTTC). The resulting cDNA was cloned into the TA cloning vector (Invitrogen). Mouse cDNA was subcloned into EYFP-C1 (Clontech) and pGEX-4T-2 (Amersham) vectors.

At that time, BLAST analyses did not reveal any available partial or full-length *Xenopus *cDNA clone (X-ASAP). We PCR-screened primary and secondary DNA pools of *X. laevis *tadpole stage 24 (Library N° 725, RZPD, Germany) using primers derived from the AW 764609 and AW 764964 sequences that contained a partial cDNA X-ASAP sequence (X2F: 5'-AAAGCAGCATTTGAGGCATGG-3' and X1R: 5'-TGATAGCGGTTATAGTATCGTTC-3'). We isolated a single partial ORF clone (XL1) that also lacked the 3' end. However, this clone was overlapping with XL402j02  and was kindly provided by the NIBB (NBRP *Xenopus *ANE library) [65]. A full cDNA clone was reconstructed by PCR, cloned into a TOPO-TA cloning vector and fully sequenced. *Xenopus *cDNA was subcloned into EYFP-C1.

### Generation and affinity-purification of polyclonal mouse ASAP antibodies

Polyclonal rabbit sera to the full-length mouse protein were raised against the corresponding GST fusion protein purified from bacteria *E. coli *BL21RP^+^. The antibodies were affinity purified on a GST column followed by a GST-mASAP fusion protein affinity column. Antibodies were routinely used at 1:500 for immunofluorescence and 1:5000 for western blots.

### Tissue expression

Two human tissue northern blots (MTN I and MTN II, Clontech) were probed with a random-primed [32P]dCTP-labelled ASAP cDNA. Hybridizations were performed at 42°C and the blots were washed at high stringency according to the manufacturer's instructions, and then exposed in a PhosphorImager cassette for one week.

To assay mRNA transcripts expression in mouse, total cellular RNA was extracted from normal adult mouse tissues using the GenElute Total Mammalian Total RNA kit (Sigma-Aldrich) following the manufacturer's instructions. Total RNA were treated with DNAse (DNA-free kit from Ambion) as indicated by the manufacturer. cDNA synthesis and PCR amplification were performed with Superscript one-step RT-PCR (Invitrogen), using 200 ng of total RNA. Each element of a primer pair was chosen in different exons to discriminate with possible contaminations by genomic DNA (mFIS2F: 5'-AAGTGAAGACAGAAACACGAAG-3'; mFIS1R: 5'-CTGTGCATTTCATGTAAATACAC-3'), and amplification was performed for 30 cycles during which the exponential phase of PCR amplification was maintained. GAPDH cDNA was amplified for 24 cycles with the primers GAPDH1: 5'-GACCACAGTCCATGCCATCACT-3' and GAPDH2: 5'-TCCACCACCCTGTTGCTGTAG-3'. Ten microliters of PCR products were analyzed on a 1% ethidium bromide-stained agarose gel.

To assay mASAP protein expression in mouse, 1 to 2 mg of each tissue were directly crushed and lysed 2 h in (50 mM Tris-HCl, 2% SDS, 50% glycerol, 1% β-mercapto-ethanol) buffer supplemented with protease and phosphatase inhibitors). After 15 min centrifugation at 13000 rpm, supernatant was collected and proteins were analyzed by western-blot as described below.

### Cell culture and transfections

All cell culture media and additives were obtained from Sigma. Human U-2 OS and Mouse NIH3T3 cells were routinely grown at 37°C in a 5% CO2 atmosphere in DMEM (Sigma) supplemented with 10% FBS, L-glutamine, and penicillin/streptomycin. Where indicated, cells were synchronized by a thymidine-block (2 mM) 24 h, released and analyzed at S, G2 or M phases. *Xenopus *XL2 cells were grown in L-15 medium as described [66]. U-2 OS and NIH3T3 cells were transfected using JetPei (Polyplus), while XL2- cells were transfected using the Amaxa-nucleofector system (solution T, program T-20), following the manufacturer's instructions. Human ASAP siRNA sequence and transfection procedure are described in [22].

### Antibodies

Antibodies were used at dilutions or concentrations stated by the manufacturer, for both western blotting and immunofluorescence microscopy, unless indicated otherwise. Primary antibodies used were anti-α-tubulin (DM 1A, Sigma), anti β-tubulin (TUB 2.1, Sigma), anti-γ-tubulin (GTU-88, Sigma), anti-GFP (Zymed); mouse polyclonal anti-ASAP (1/500 for immunofluorescence and 1/3000 for western blotting). Secondary antibodies used for immunofluorescence were coupled to Alexa Fluor 488-, Alexa Fluor 546- or Alexa Fluor 642-conjugated goat anti-mouse or anti-rabbit IgG (1/1000, Molecular Probes). Secondary antibodies used for the immunoblots were peroxidase-conjugated goat anti-mouse or anti-rabbit (1/5000, Zymed). Hoechst 33258 was purchased from Sigma.

### Cell extracts and Western blot analysis

Cells were washed with ice-cold PBS, scraped off the plate, and resuspended in ice-cold lysis buffer (50 mM Tris pH8.0, 120 mM NaCl, 5 mM EDTA, 0.5% NP40) supplemented with 1 mM DTT and a protease inhibitor cocktail tablet (Roche) and phosphatase inhibitors. After 15 min on ice, lysed cells were centrifuged at 13000 rpm for 10 min at 4°C. Ten to 15 mg of murine brain or testis were directly lysed in 1 ml of Laemmli buffer (50 mM Tris pH8.0, 2% SDS, 10% glycerol, 1% β-Mercaptoethanol). Protein concentrations in the cleared lysate were determined using a Bradford assay, and equal amounts were loaded on SDS-PAGE gels. Separated proteins were transferred to nitrocellulose membrane (Whatman Schleicher and Schuell) and were detected by various antibodies and visualized by enhanced chemiluminescent reagents (Supersignal West-PicoPico, Pierce Chemical).

### Immunofluorescence microscopy

Cells grown on coverslips were fixed either by incubation in 4% paraformaldehyde in MTSB (Microtubule Stabilization buffer: 100 mM PIPES, 1 mM EGTA, 4% PEG 8000, pH 6.9) 10 minutes at room temperature followed by 0.5% Triton X-100/MTSB for 5 min (PAF/MTSB fixation) or by incubation in formaldehyde 3.6% in PHEM (60 mM Pipes, 25 mM Hepes, 10 mM EGTA, 2 mM MgCl2, pH 6.9) for 10 minutes followed by methanol 1 minute at room temperature (F/PHEM/methanol fixation). Fixed cells were incubated for 1 h at 37°C with the primary antibody, and 30 min at 37°C with the secondary antibody. All antibodies were diluted in PBS/3% BSA. Coverslips were mounted using Gel-Mount (Biomedia). Images were acquired on a Leica DM6000B fluorescence or a confocal Leica TCS SP2 microscopes using CCD cameras and subsequently processed by the Metamorph or LCS LEICA confocal softwares, respectively.

### Cryosection and Immunofluorescence

Testis tissues were washed twice in PBS, fixed in 3.7% (vol/vol) paraformaldehyde/PBS for 18 h at 4°C, washed twice in PBS, and incubated overnight in 30% (wt/vol) sucrose/PBS. Samples were embedded in OCT medium (Tissue-Tek) and frozen at -70°C, and serial 12 μm sections were cut with a cryostat (Leica). After drying, the sections were rehydrated in PBS for 5 min. Tissue sections processing for immunofluorescence using the polyclonal mASAP rabbit serum (used at a 1/500 dilution) were as described [67].

### Culture of mouse cortical neural stem cells and immunocytochemistry

The protocol used was described in Milhavet *et al*. [68] with minor modifications: during proliferation cells were cultured in a medium containing DMEM-F12 (Invitrogen Life Technologies, Cergy Pontoise, France) with modified N2 supplement and 25 ng/ml of Basic fibroblast growth factor (βFGF, Abcys, France). βFGF was added daily and medium changed every two days. Undifferentiated cells cultured in presence of βFGF expressed primarily nestin, an intermediate filament protein expressed in neural stem cells and progenitors, whereas β-III tubulin, a neuronal marker and GFAP, an astrocytic marker, were not. After two passages, cells at 80% confluence were differentiated by removal of βFGF in a culture medium containing 50% of DMEM-F12 with modified N2 supplement and 50% Neurobasal with B27 supplement (Invitrogen Life Technologies, Cergy Pontoise, France). During differentiation the medium was changed every two days. Cells were fixed in 4% paraformaldehyde plus 0.15% picric acid in PBS, and standard immunocytochemical protocols followed before observation with a Zeiss Axiovert 200 M microscope. The following primary antibodies were used: ASAP rabbit antibody at 1:500, β-tubulin type III (Tuj1) monoclonal antibody at 1:1000 (Covance Research Products, Berkeley, CA, USA) for specific detection of neuronal cells, and rabbit glial fibrillary acidic protein (GFAP) at 1:1000 (Dakocytomation, Trappes, France) for specific detection of glial cells. Appropriate fluorescence-tagged secondary antibodies (AlexaFluor 488 and 555, Invitrogen Life Technologies, Cergy Pontoise, France) were used for visualization. DAPI was used for nuclear counterstaining.

## Authors' contributions

SR and DG conceived, designed the study and wrote the paper. All authors carried out the experiments and participated in interpreting the data, and read and approved the final manuscript.
